# Knock-out of the critical nitric oxide synthase regulator DDAH1 in mice impacts amphetamine sensitivity and dopamine metabolism

**DOI:** 10.1007/s00702-023-02597-7

**Published:** 2023-02-16

**Authors:** Alena A. Kozlova, Elena Rubets, Magdalini R. Vareltzoglou, Natalia Jarzebska, Vinitha N. Ragavan, Yingjie Chen, Jens Martens-Lobenhoffer, Stefanie M. Bode-Böger, Raul R. Gainetdinov, Roman N. Rodionov, Nadine Bernhardt

**Affiliations:** 1 Department of Psychiatry and Psychotherapy, University Hospital Carl Gustav Carus, Technische Universität Dresden, 01307 Dresden, Germany; 2 Division of Angiology, Department of Internal Medicine III, University Center for Vascular Medicine, Technische Universität Dresden, 01307 Dresden, Germany; 3 Department of Physiology & Biophysics, University of Mississippi Medical Center, Jackson, MS 39216 USA; 4 Institute of Clinical Pharmacology, Otto-Von-Guericke University, Magdeburg, Germany; 5 Institute of Translational Biomedicine and Saint-Petersburg University Hospital, Saint-Petersburg State University, 199034 Saint-Petersburg, Russia

**Keywords:** DDAH1, Knock-out mice, Behaviour, Nitric oxide, Dopamine, Amphetamine

## Abstract

The enzyme dimethylarginine dimethylaminohydrolase 1 (DDAH1) plays a pivotal role in the regulation of nitric oxide levels by degrading the main endogenous nitric oxide synthase inhibitor asymmetric dimethylarginine (ADMA). Growing evidence highlight the potential implication of DDAH/ADMA axis in the etiopathogenesis of several neuropsychiatric and neurological disorders, yet the underlying molecular mechanisms remain elusive. In this study, we sought to investigate the role of DDAH1 in behavioral endophenotypes with neuropsychiatric relevance. To achieve this, a global DDAH1 knock-out (DDAH1-ko) mouse strain was employed. Behavioral testing and brain region-specific neurotransmitter profiling have been conducted to assess the effect of both genotype and sex. DDAH1-ko mice exhibited increased exploratory behavior toward novel objects, altered amphetamine response kinetics and decreased dopamine metabolite 3,4-dihydroxyphenylacetic acid (DOPAC) level in the piriform cortex and striatum. Females of both genotypes showed the most robust amphetamine response. These results support the potential implication of the DDAH/ADMA pathway in central nervous system processes shaping the behavioral outcome. Yet, further experiments are required to complement the picture and define the specific brain-regions and mechanisms involved.

**Supplementary Information:**

The online version contains supplementary material available at 10.1007/s00702-023-02597-7.

## Introduction

Nitric oxide (NO) is a gaseous molecule with multifaceted physiological and pathophysiological functions within the central nervous system (CNS) (Esplugues 2002). NO is the product of l-arginine oxidation to l-citrulline in a two-step redox reaction catalyzed by nitric oxide synthases (NOS). Physiological levels of NO have been shown to fine-tune food intake, sleep, learning and memory, hormone release, synaptic transmission, neurosecretion and thermal regulation, whereas disturbances in NO levels have been associated with neuroinflammation and nitrosative stress (Nelson et al. 1997; Calabrese et al. 2007; Chung and Park 2008). Due to the core nature of these processes, it is no wonder that imbalanced NO synthesis has been reported in a series of neuropsychiatric disorders, including schizophrenia (Hoffer et al. 1954; Flatow et al. 2013; Nasyrova et al. 2015), stress-induced anxiety (Kumar and Chanana 2017), affective disorders (Zhou et al. 2018), and attention deficit hyperactivity disorder (Tas et al. 2006). Genome-wide association studies in psychiatric patients have also associated genetic variation in NOS genes with major psychiatric conditions and have proposed a common genetic overlap between these disorders (Freudenberg et al. 2015). In support of the above clinical data, animal models further pinpoint that many behavioral domains, including impulsivity, hyperactivity, aggression, anxiety, depression-like symptoms as well as cognitive performance, are influenced by manipulating NO levels (Nelson et al. 1995; Weitzdoerfer et al. 2004; Trainor et al. 2007; Wultsch et al. 2007; Tanda et al. 2009; Zhang et al. 2010; Gao and Heldt 2015). Furthermore, interaction partners of NOS have also been associated with psychiatric symptom manifestation, with NOS 1 adaptor protein (NOS1AP) polymorphisms being a prominent example of schizophrenia and bipolar disorder risk factor (Freudenberg et al. 2015). Therefore, both NO and its regulators are key components in the pathology of neuropsychiatric disorders.

Dimethylarginine dimethylaminohydrolase 1 (DDAH1) plays a central role in the homeostatic control of NO by metabolizing the endogenous NOS inhibitors monomethylarginine (L-NMMA) and asymmetric dimethylarginine (ADMA) (Vallance et al. 1992; Cardounel and Zweier 2002). Overexpression of *Ddah1* depletes ADMA from blood plasma and tissues and thus increases NO bioavailability in murine models (Dayoub et al. 2003, 2008; Schwedhelm et al. 2009). In contrast, knock-out of *Ddah1* led to elevated levels of ADMA and decreased NO in tissues and plasma (Hu et al. 2011; Zhao et al. 2021). A pathophysiological role of DDAH1 via the ADMA/NO pathway has already been established in non-CNS conditions, including cardiovascular (Leiper et al. 2007; Dayoub et al. 2008; Hu et al. 2011) and renal disease (Vallance et al. 1992; Tomlinson et al. 2015), angiogenesis, vascular permeability and wound healing (Smith et al. 2003; Achan et al. 2005; Jacobi et al. 2005; Konishi et al. 2007; Wang et al. 2021), vascular remodeling (Rodionov et al. 2010, 2022; Kopaliani et al. 2021) and insulin sensitivity (Sydow et al. 2008); however, its implication in the CNS is not yet extensively studied. So far, studies have shown that DDAH1 is widely expressed in the postnatal CNS, specifically in neurons, astrocytes and the endothelial cells of blood vessels (Tran et al. 2000; Kozlova et al. 2022), suggesting that DDAH1 may be a principal NO regulator in the brain.

Recently, the DDAH/ADMA/NO axis, primarily concerning the DDAH1 substrate ADMA, has been investigated in the context of neuropsychiatric disorders. Systemic ADMA concentrations were found to be elevated in patients with schizophrenia (Das et al. 1996; Celik et al. 2011; Zincir et al. 2014; Telo and Gurok 2016; Yang et al. 2016; Yu et al. 2019; Ustundag et al. 2020; Braun et al. 2021), bipolar disorders (Aykut et al. 2012; Ustundag et al. 2020; Braun et al. 2021), attention deficit hyperactivity disorder (Doneray et al. 2022) and depression (Selley 2004; Mcevoy et al. 2013; Baranyi et al. 2014, 2015; Ogłodek 2017; Braun et al. 2021; Loeb et al. 2021), accompanied by decreased plasma NO levels (Das et al. 1996; Selley 2004; Aykut et al. 2012; Ustundag et al. 2020; Doneray et al. 2022). Furthermore, treatment administration normalized plasma ADMA levels in schizophrenia (Das et al. 1996; Zincir et al. 2014; Yu et al. 2019), bipolar disorders (Tiryaki et al. 2017), and attention deficit hyperactivity disorder (Doneray et al. 2022). However, since these studies have focused exclusively on changes in peripheral ADMA levels in patients, it is currently unknown whether modifications of DDAH1 expression and activity causally link to behavioral phenotypes. Therefore, using global DDAH1 knock-out (DDAH1-ko) mice, we sought to address this issue and investigate the role of reduced DDAH1 expression in modulating behaviors that function as endophenotypes of neuropsychiatric disorders.

## Materials and methods

### Animals

Global DDAH1-ko mice on the C57BL/6J background were used as described (Hu et al. 2011). Briefly, the DDAH1^flox/flox^ mice were crossed with protamine (Prm)-cre mice (129-Tg(Prm-cre)58Og/J, Jackson Laboratory) and the DDAH1 gene was deleted in the sperm of the male double heterozygote Prm-cre/DDAH1^flox/+^ mice. When these male mice were crossed with wild-type female breeders, DDAH1^+/−^ mice were generated. The homozygote global DDAH1^−/−^ was generated by the inbreeding of the heterozygotes. Genotyping was performed by a polymerase chain reaction from tail samples using the following primer’s pairs: forward 5´-AAT CTG CAC AGA AGG CCC TCA A-3´ and reverse 5´-GGA GGA TCC ATT GTT ACA AGC CCT TAA CGC-3´ for the wild type allele and forward 5´-TGC AGG TCG AGG GAC CTA ATA ACT-3´ and reverse 5´-AAC CAC ACT GCT CGA TGA AGT TCC-3´ for the knock-out allele. Animals were housed at constant temperature (23 ± 2 °C), humidity (50 ± 10%), and a 12 h light–dark cycle (light on at 06:00 am). Experimental mice were housed in mixed genotype groups (4–5 animals per cage) with food and water ad libitum. Experiments were conducted on four birth cohorts of 12–14-week-old mice. A total of 37 DDAH1-ko and 36 wild-type (wt) mice were used. These groups included both males (DDAH1-ko: *n* = 24; wt: *n* = 23) and females (DDAH1-ko: *n* = 13; wt: *n* = 13).

### Behavioral experiments

13 DDAH1-ko (8 male, 5 female) and 13 wt (8 male, 5 female) animals were used to perform a battery of behavioral tests over 3 weeks. Experiments were conducted during the light phase (between 8:00 a.m. and 6:00 p.m.) by a female investigator blind to the animal’s genotype. All mice were subtracted to the same order of experiments. In preparation for behavioral testing, mice were handled twice daily for 5 days. During the course of the experiment, body weight and food intake were continuously assessed. All apparatus was cleaned with 70% ethanol between each animal. Data were acquired using ManyCam (Visicom Media Inc.), analyzed with EthoVision XT 11.5 software (Noldus Information Technology), and confirmed with manual inspection of the video material when required. The behavioral tests were selected for their relevance to neuropsychiatric disorders and are regularly used in our lab (Komada et al. 2008; Can et al. 2012a, b; Angoa-Pérez et al. 2013; Hadar et al. 2016, 2020; Huang et al. 2016; Meyerolbersleben et al. 2020). They were performed in the following sequence:

*Neurological deficit score test* was performed to assess the general neurological condition of experimental animals (Huang et al. 2016). The test included 9 sub-tasks. Briefly, mice were placed in a round arena (diameter 140 cm) and the latency (s) to exit a 30 cm-diameter circle was measured. Gross motor activity and coordination, loss of startle behavior and seeking behavior were additionally judged. Finally, beam walking on 3 cm, 1.5 cm and 1 cm-width beams placed 40 cm above the floor over a 50 cm distance was assessed. Wire hanging was also tested on a round (0.5 cm) and a square wire (1 cm). A 15-point scale was used, where 0 indicates lack of impairments and 15 indicates severe neurological dysfunction.

*Tail suspension test* was performed to assess depressive-like behavior and active coping strategies (Can et al. 2012b). Each mouse was suspended 30 cm above the table surface with a 5 cm long tape on a wooden stick. The behavior was recorded for 6 min and the extent of immobility (s) was measured. Due to the aversive nature of the test, the animals were allowed to rest for 24 h to exclude immediate stress interference with the following task.

*Open field test* was used to evaluate basal locomotor activity and innate anxiety-like behavior in an unfamiliar square arena (45 cm × 45 cm × 45 cm high). Each mouse was placed in the border zone facing the wall and allowed to explore freely for 10 min. Horizontal locomotor activity was estimated by measuring the total distance moved (m) and velocity (cm/s), while anxiety-related behavior was assessed by the time spent in a center zone (s) defined by the experimenter (15 cm × 15 cm).

*Novel object recognition test* was conducted to assess object interaction and recognition memory. The task consisted of three phases: habituation, familiarization, and test phase. Open field analysis was conducted before the novel object recognition test, and thereby considered as habituation to the arena environment. On the same day at familiarization trial, the animals were exposed to two identical objects placed at opposite corners of the arena and allowed to freely explore for 10 min. After 24 h, the test phase (retrieval) followed, during which one of the initial objects was removed and replaced by a novel (different in form and color) randomly assigned to the left or right side of the box. Again, mice were placed in the arena for 10 min, and activity was recorded. Familiar and novel object exploration was analyzed using nose-tracking in the vicinity of the objects. Exploration was defined as sniffing or nose-touching the object, while climbing, sitting on the object or touching it with the body were excluded. Time spent for object exploration (s), time exploring the novel object (s) and time exploring the familial object (s) on day 2 were measured.

*Social interaction test* was performed to assess the sociability of animals toward an unknown age and gender-matched “actor” mouse. Actor mice were raised and kept in a separate room except for the test phase. Before the test, the actor mice were trained to be placed into a restrainer made of wired metal (20 × 8 cm) for 3 days to reduce the effects of restrained anxiety. In parallel, the test mice were familiarized with the empty restrainer by placing it in one corner of the familiar open field arena. On the test day, an actor mouse was placed in the restrainer positioned in the arena, while a test mouse was placed in the opposite corner facing the wall. The test mouse was allowed to explore freely for 10 min. Frequency of social contacts and time spent in social exploration (s) defined as sniffing or nose-touching the actor was measured.

*Sucrose preference test* was performed according to previously reported protocols (Meyerolbersleben et al. 2020). During the experiment, mice were individually housed with water and food ad libitum. Two 250 ml bottles were filled with water, weighed and placed next to each other on one side of a wire rack. To establish baseline water consumption, each bottle was first measured for 2 days containing water (baseline days). On the third day, water in one of the bottles was changed to a 2% sucrose solution and consumption was measured for the next 3 days (test days). Bottles were changed daily to prevent side preferences. Consumption was calculated as the daily reduction of weight for each bottle, and thus sucrose consumption (ml) and water consumption (ml) were measured.

*Elevated plus maze test* was performed to assess anxiety-like behavior as previously described (Komada et al. 2008). Briefly, the experiment was conducted in a maze made from dark opaque PVC, which consisted of two opposing open arms (*L* 33 cm, *W* 5 cm, *H* 15 cm, 300 Lux) and two opposing closed arms (*L* 33 cm, *W* 5 cm, *H* 15 cm, 10 Lux) departing from a mid-area (7 cm × 7 cm). The plus-shaped maze was placed 70 cm above the floor. Each mouse was placed in the center square of the maze facing one of the open arms. Acquiring was continued for 10 min. Time spent (s) and the number of entries in opened and closed arms were measured.

*Forced swim test* was conducted to assess active-coping behavior, which can also serve as a measure for depressive-like behavior estimation (Can et al. 2012a). Animals were placed individually in a cylinder (*H* 25 cm, diameter 17 cm) filled with water at 23 ± 1 °C and activity was recorded for 6 min. Water was changed after each mouse. Immobility time (s) was measured. Due to the aversive nature of the test, the animals were allowed to rest for 24 h to exclude immediate stress interference with the following task.

*Nestled shredding test* was used to assess persistent and repetitive behavior (Angoa-Pérez et al. 2013). Animals were placed individually in an unfamiliar standard housing cage with a cotton pad (1 cm × 1 cm, 2 g in weight) located in the opposite corner. The test was carried out for 30 min without food and water, and then the remaining unshredded pad was weighted for analysis.

*Marble burying test* was conducted to assess persistent and repetitive behavior (Angoa-Pérez et al. 2013). Mice were placed in an unfamiliar standard housing cage filled with unscented bedding material to a depth of 5 cm, where 20 glass marbles (4 columns and 5 rows) were placed. The test was performed for 30 min without food and water, and then the number of buried marbles (at least 2/3 covered with bedding material) was counted.

*Amphetamine-induced locomotion activity test* was conducted to analyze the effect of amphetamine on motor activity. First, animals were injected with 0.1 ml of saline [intraperitoneal injection (i.p.) 0.9% solution of sodium chloride (B. Braun Melsungen AG, Germany)], placed individually in the open field and recorded for 30 min. Then, mice were injected with a single dose of amphetamine 5 mg/kg bodyweight [i.p., freshly prepared Dexamfetaminhemisulfat (LIPOMED GmbH, Germany) in 0.9% saline] and were recorded for another 60 min. Horizontal locomotor activity was assessed as distance moved (m) over 5 min time-bins (Hadar et al. 2020). Stereotypic behavior was also analyzed as events of continuous rearing, continuous sniffing, frequent sniffing at body and genitals, frequent biting, gnawing or licking and frequent head swaying/head bobbing (Hadar et al. 2016).

Following the behavioural assessment the mice were sacrificed as described below and their brains were used for macroscopic evaluation of brain morphology and immunohistochemistry.

### Tissue collection

Mice were deeply anesthetized intraperitoneally with a mixture of 100 mg/kg ketamine and 10 mg/kg xylazine. For high-performance liquid chromatography (HPLC), western blot, real-time reverse transcription–polymerase chain reaction (RT–PCR) and DDAH activity assay mice were decapitated, brains were immediately extracted and frozen with methylbutane at − 20 °C in liquid nitrogen and stored at − 80 °C. For immunohistochemistry, mice were transcardially perfused, brains were dissected and post-fixed in 4% paraformaldehyde overnight, cryoprotected in 20% sucrose in phosphate buffer solution (PBS) for up to days, frozen in methylbutane at − 40 °C and then stored at − 80 °C. Coronal sections of 40 µm thickness were cut on a freezing microtome (Leica CM1850) and stored in antifreeze medium (25% glycerol, 25% ethylene glycol in PBS) at − 20 °C until further processing.

### Measurement of neurotransmitters levels (HPLC)

An independent set of 14 DDAH1-ko (8 males, 6 females) and 13 wt (7 males, 6 females) animals that had not undergone any behavioral experiments was used for HPLC analysis to exclude inferences of the amphetamine challenge with the neurotransmitter measurements. Micro-punches of brain tissue were homogenized in 250 µl of 0.1 N perchloric acid using a manual homogenizer (Polytron PT 1200 E, Kinematica, Luzern, Suisse) that was applied 3 × 10 s at maximum speed on ice. After protein quantification (Pierce™ 660 nm Protein Assay; Multiskan™ FC, ThermoFisher Scientific™, Germany), the homogenates were centrifuged for 15 min at 13,000*g* and 4 °C and used to detect noradrenaline (NOR), dopamine (DA) with its metabolite 3,4-dihydroxyphenylacetic acid (DOPAC). Monoamines were separated on a column (ProntoSIL 120-C18-SH, VDS Optilab, Germany) at a flow rate of 0.25 ml/min and a column temperature of 32 °C before electrochemical detection (Dionex™ Coulochem™ III, ThermoFisher Scientific™, Germany). Mobile phase (pH 3.48 of 80 mM NaH2PO4*H2O, 0.5 mM EDTA-Di-Na, 0.72 mM sodium 1-octanesulfonate, H3PO4 and 2-Propanol). Sample peak areas were measured via Labsolutions integrator system (LCsolution Version 1.24 SP1 Integration Time Program).

### Western blot

Whole brain hemispheres from 5 DDAH1-ko and 5 wt mice were used for model validation using western blot and RT–PCR analyses. Left and right hemispherse were randomly assigned to protein or mRNA quantification. Brain tissue samples were homogenized (2 × 20 s at 6500 rpm) and sonicated (3 × 10 s burst, 60% amplitude, 4 °C) in ice-cold RIPA buffer (0.15 M NaCl, 0.05 M Tris, pH 8.0, 1% NP-40, 0.5% DOC, 0.1% SDS) containing a protease inhibitor mixture (Mini-complete Protease Inhibitor Cocktail Tablets; Roche Diagnostics-Applied Science, Mannheim, Germany). Total protein concentration in cell lysates was determined using a Pierce BCA Protein Assay Kit (Thermo Scientific™, USA). Protein extracts (20 μg/μL) were diluted with Laemmli buffer (0.25 M Tris–HCl, 8% SDS, 40% glycerol, 0.2 mg/mL bromophenol blue, 20% β-mercaptoethanol), run in 10% SDS polyacrylamide gel by electrophoresis (100 V for 20 min, 150 V for 1 h) and transferred by wet transfer (100 V for 1 h at 4 °C) onto ethanol-activated PVDF membranes (PVDF Western Blotting Membrane, Roche, Mannheim, Germany). Membranes were blocked in 3% blocking buffer (3% milk, TBS: 50 mM Tris–HCl, pH 7.4, 150 mM NaCl) at room temperature (RT) for 1 h. The membranes were incubated overnight at 4 °C in an antibody solution containing TBST (50 mM Tris-HCI pH 7.4, 150 mM NaCl, 0.2% Tween 20), 2% milk and 1:1000 rabbit anti-DDAH1 antibodies (MERCK, HPA006308). After washing three times in TBST for 10 min, membranes were further incubated with a 1:2000 horseradish peroxidase-conjugated goat anti-rabbit antibody solution (Jackson, 035-144) or 1:30,000 horseradish peroxidase-conjugated anti-β-actin antibody solution (Sigma Aldrich, A3854), followed by washing in TBST for 15 min three times. Immune-reactive bands were detected by Roche “Lumi-Light Western Blotting Substrate” (Roche, Germany) and visualized using PeqLab Fusion Fx7 Imaging System (Peqlab, Germany). Bands were quantified by densitometric analysis using ImageJ software.

### RNA isolation and RT-PCR

Total mRNA isolation was carried out using the RNeasy Plus Mini Kit (Qiagen, Germany). The RNA concentration, ratios A260/A280 and A260/A230 were directly measured by Multi-Detection Microplate Reader (The Synerg HT BIO-TEK, Vermont, USA) with Bio Tek Gen5 software. 0.5 µg of total mRNA was reverse transcribed to cDNA using the High Capacity cDNA Reverse Transcription Kit (Applied Biosystems, USA). All kits were applied according to the manufacturer’s instructions. The amplification of the cDNA templates for quantification was performed with the Maxima SYBR Green/Rox kit (Thermo Scientific™, Germany). cDNA was first denatured for 10 min at 95 °C, followed by 40 cycles of denaturation for 15 s at 95 °C and annealing and extending for 60 s at 60 °C. Data were analyzed using 7500 software (version 1.3.1, Applied Biosystems, Foster City, USA) and expressed as a ratio to levels of hypoxanthine–guanine–phosphoribosyltransferase (HPRT) mRNA and plotted as fold-change to wild type controls. The primer pairs used in the qPCR for amplification of the respective cDNA sequences were designed using the Primer-BLAST tool (https://www.ncbi.nlm.nih.gov/tools/primer-blast/). The following primer pairs were used: murine DDAH1 (forward 5´-CTACGCAGTCTACAGT, reverse 5´-TCATAACGATCACTCA), murine HPRT (forward 5´-CTTGCTGACGATTAC, reverse 5´-ATCCAACACTAGGTCC). All primers were synthesized by Biomers.net (Ulm, Germany).

### DDAH activity

The protocol for the measurement of DDAH activity in whole brain tissue lysates from 5 DDAH1-ko and 5 wt mice was modified based on published methods (Maas and Strubelt 2006; Martens-Lobenhoffer et al. 2012; Burdin et al. 2016). A total of 40 mg of tissue from each sample was weighed and homogenized in 1 ml of RIPA buffer (0.15 M NaCl, 0.05 M Tris (pH 8.0), 0.5% sodium deoxycholate, 1% NP40, 0.1% SDS) containing protease inhibitor and 1 mM PMSF at 6500 rpm, twice for 20 s. Tissue homogenate was incubated on ice for 10 min and later centrifuged at 14 000 × g for 10 min at 4 °C. The cleared lysate was transferred to a fresh tube and the protein concentration was measured (Pierce™ BCA Protein Assay Kit). A total of 100 µL of cleared lysate was diluted in 1 mL of phosphate buffered saline incubation buffer (NaH_2_PO_4_ adjusted to pH 8.0 with 1 M H_3_PO_4_) containing a final concentration of 800 µM stable isotope labelled ADMA (#DLM-7476-0, Cambridge Isotope Laboratories). The mixture was incubated at 37 °C for 1 h and flash frozen in liquid nitrogen to stop the reaction. The sample was analyzed by HPLC–MS–MS, measuring the resulting labelled citrulline formation from the metabolism of labelled ADMA and the results were normalized to the total protein content of the corresponding samples.

### Immunofluorescence

Staining of brain sections was conducted based on a free-floating sections protocol on series of every 12th section. Primary washing was done for 3 × 15 min in PBS followed by blocking with 10% normal goat serum (NGS, ab7481, Abcam) and 0.2% Triton X-100 in PBS for 2 h at RT. Afterward, sections were incubated overnight at 4 °C with primary antibodies 1:1000 (DDAH1, HPA006308, MERCK) diluted in 3% NGS and 0.2% Triton X-100 in PBS. At the next day, sections were washed for 3 × 15 min in PBS followed by incubation with secondary antibodies diluted 1:1000 in 3% NGS and 0.2% Triton X-100 in PBS for 2 h at RT. Next the sections were washed 3 × 15 min in PBS and nuclei were stained for 10 min with 4′,6-diamidino-2-phenylindole (DAPI) diluted 1:5000 in PBS. After the last washing for 10 min, sections were mounted with Mowiol (MERK, no. 475904) on glass slides (SuperFrost Ultra Plus, Thermo Scientific). Samples were visualized and analyzed using Zeiss Observer Z.1 (ApoTome II).

### Statistical analysis

For the dependent variable ‘DDAH1 mean fluorescence intensity’, the main effect of genotype was analyzed using Mann–Whitney *U* test due to non-normal data distribution. The variables ‘band density ratio’, ‘relative mRNA’ and ‘DDAH1 activity’ were analyzed using two sided *t* test with adjustment for non-equal distribution. Gross morphology and behavioral data were tested for normal distribution and equal variance and analyzed with two-way analysis of variance (ANOVA) for the main factors genotype (wt, DDAH1-ko) and sex (male, female). The preference index in the novel object recognition test was calculated from the exploration time on day 2 as familiar/(familiar + novel) × 100. Sucrose Preference Index from the sucrose preference test was calculated according to the formula Sucrose Preference = *V*(sucrose solution)/[*V*(sucrose solution) + *V*(water)]. For each dependent variable, namely, ‘absolute consumption’ and ‘sucrose preference’, we conducted a repeated-measures analysis of variance (RM-ANOVA) with genotypes, sex and testing days. Locomotor activity in the open field after amphetamine treatment was analyzed using RM-ANOVA of distance moved in 5-min time. HPLC analysis was conducted using multivariate analysis of variance (MANOVA) with genotype, sex and region of interest set as factor with Bonferroni correction. Main analyses were performed using SPSS Statistics (version 27.0, IBM, Armonk, NY, USA). For ANOVA models all data are presented as mean (M) ± standard deviation (SD), and full output tables of statistical results can be found in Tables S1–2. Statistical significance was set at *p* < 0.05. Graphs were made with PlotsOfData (Postma and Goedhart 2019).

## Results

### DDAH1-ko mice lack DDAH1 expression and exhibit normal physiological and morphological features

**Fig. 1:**
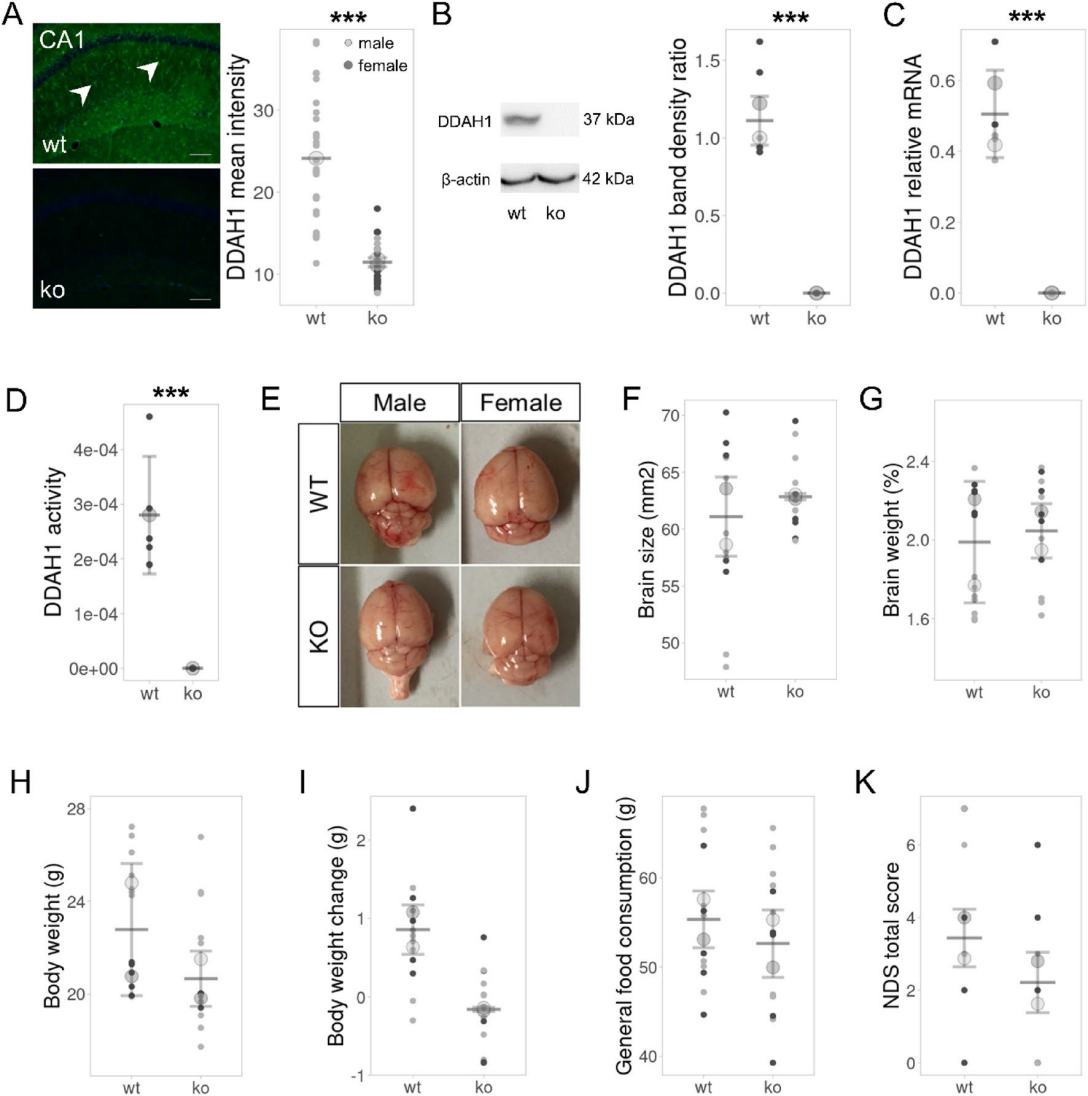
Lack of DDAH1 was not associated with major physiological, morphological, and neurological phenotypes. **A** Immunofluorescent analysis revealed the absence of DDAH1 expression in the hippocampus (CA1) of DDAH1-ko mice; 3 sections from each wt: *n* = 6, DDAH1-ko: *n* = 6; Mann–Whitney *U* test. **B**, **C** Western blot and RT-PCR analysis confirmed the above result. **D** Total DDAH activity was reduced in DDAH1-ko animals. For B–D wt: *n* = 5, DDAH1-ko: *n* = 5; *t* test. **E**–**G** Extracted brains did not differ between genotypes in morphology, size, and weight; wt: males: *n* = 8, females: *n* = 5, DDAH1-ko: males: *n* = 8, females *n* = 5; two-way ANOVAs. **H** Lower body weight is observed in DDAH1-ko animals. **I** No difference has been detected in overall weight change during the course of the behavioral testing between DDAH1-ko and wt mice. **J** Total food consumption had no gender and genotype effect. **K** Quantification of the neurological deficit score (NDS) revealed no changes in general neurological condition in DDAH1-ko mice. For H–K wt: males: *n* = 8, females: *n* = 5; DDAH1-ko: males: *n* = 8, females: *n* = 5; two-way ANOVAs. Plots show mean ± SD center line and bottom and top lines; big circles represent the mean of males (light grey) and females (dark grey); small dots represent individual data points. * *p* < 0.05, ** *p* < 0.01, *** *p* < 0.001 for significant genotype effects.White arrows in (A) mark DDAH1 positive cells, scale bar—100 μm

First, to validate that DDAH1-ko mice do not have any residual DDAH1 expression, which would confound further downstream experiments, we examined the expression of DDAH1 in the brain of ko animals. DDAH1 is widely distributed in the mouse brain, with the most intense signal appearing in the striatum, cortex and hippocampus (Kozlova et al. 2022). Immunohistochemical staining and analysis of the hippocampal CA1 region, one of the most representative areas expressing DDAH1 in wild-type animals, showed the absence of DDAH1-positive cells in sections from DDAH1-ko mice (Fig. 1A, *U* = 26.000, *p* < 0.001). Overall lack of DDAH1 expression at both the transcriptional (Fig. 1C, *t* (4) = 8.449*, p* = 0.01) and the protein level (Fig. 1B, *t* (4.823) = − 2.831*, p* = 0.038) in brain samples further confirmed this finding. Finally, a loss of total DDAH activity in DDAH1-ko brain samples was observed employing an enzymatic activity assay (Fig. 1D, *t* (4) = 5.832, *p* = 0.04).

We examined the basic physiological, morphological and developmental features of DDAH1-ko mice. No gross morphological, weight and size differences have been detected in the brains between genotypes (Fig. 1E–G, Table S1). However, a sex effect on brain weight has been observed with males having smaller brains (*F* (1, 22) = 10.697, *p* = 0.003), with a significant difference in wt animals (post-hoc comparison male vs. female wt: *p* = 0.004). Body weight measured at the start of the behavioral experiments showed a significant effect of genotype (*F* (1, 22) = 4.394, *p* = 0.048) and sex (*F* (1, 22) = 7.996, *p* = 0.01, Fig. 1H, I). Post-hoc testing showed a significantly lower weight in male DDAH1-ko mice compared to male controls (*p* = 0.007) and a significant difference between male and female wt mice (*p* = 0.006). In contrast, overall weight change throughout the experimental period did not differ between groups. Food consumption has also been analyzed and no significant genotype or sex differences have been found throughout the behavioral experiments (Fig. 1J, Table S1). Finally, the total neurological deficit score, an estimate of the general neurological condition of the experimental animals, was similar in DDAH1-ko and wt mice, indicating that lack of *Ddah1* gene does not lead to profound general neurophysiological impairments (Fig. 1K, Table S1).

To sum up, we showed that DDAH1-ko mice lack DDAH1 expression and total DDAH activity in the brain. Furthermore, loss of DDAH1 resulted in lower body weight in males but no other major physiological, morphological, and neurological abnormalities.

### DDAH1-ko mice exhibited increased exploratory behavior

To evaluate alterations in more specific behaviors in DDAH1-ko mice, a battery of extensively validated and regularly used behavioral tests was employed (Komada et al. 2008; Can et al. 2012a, b; Angoa-Pérez et al. 2013; Hadar et al. 2016, 2020; Huang et al. 2016; Meyerolbersleben et al. 2020). A broad spectrum of behavioral phenotypes has been screened, including locomotor activity and innate anxiety-like behavior, exploratory behavior and recognition memory, sociability, active coping, reward function as well as persistent and repetitive behavior.

**Fig. 2:**
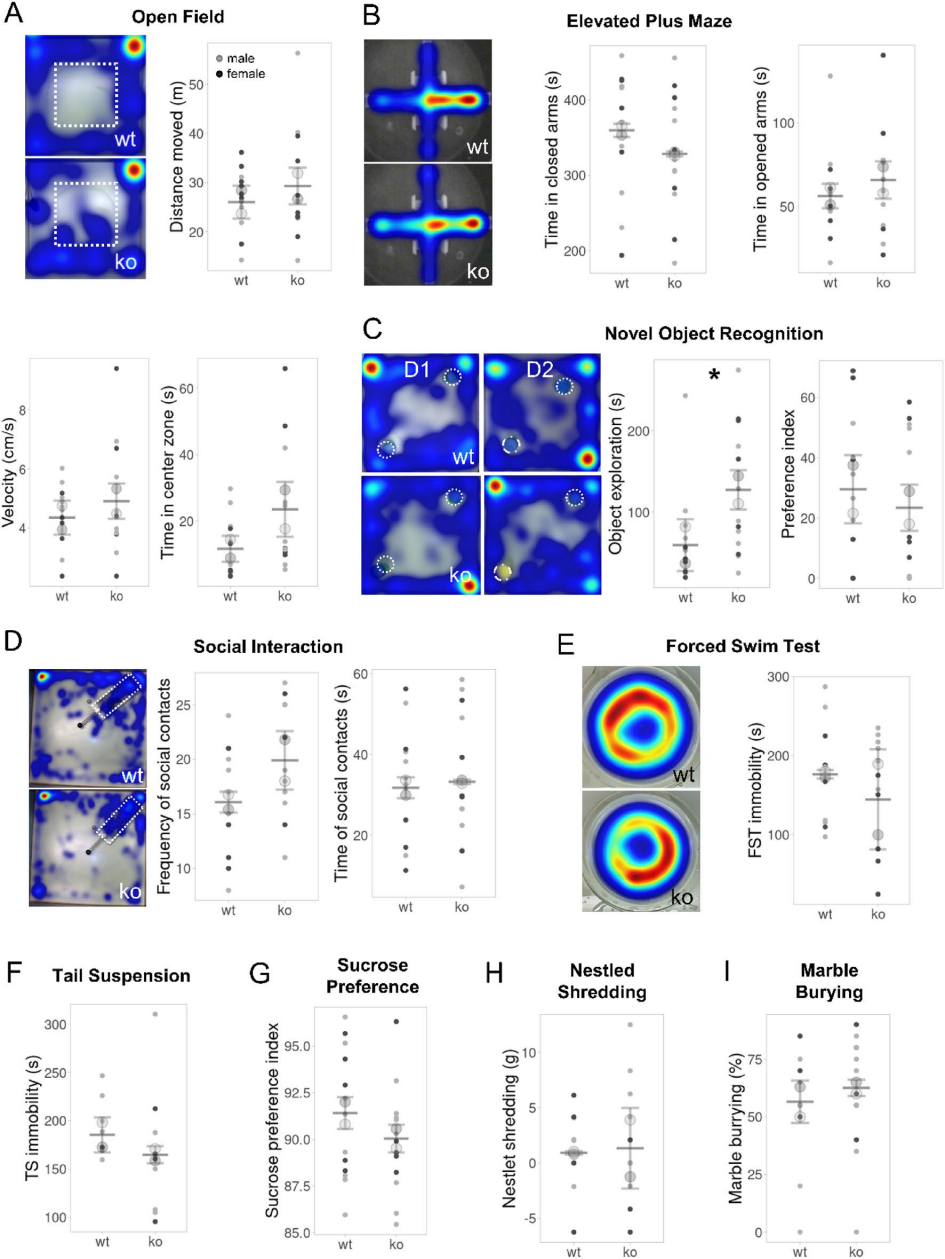
DDAH1-ko mice exhibited increased object exploratory behaviour without further behavioural alterations. **A** In the open field test, there were no differences in the distance moved, velocity and time in the center zone for both genotypes and sexes. **B** Time spent in the opened and closed arms during the elevated plus maze was also independent of these factors. **C** In the novel object recognition task, DDAH1-ko mice exhibited increased overall object exploratory activity over the 2-day assessment, though they did not exhibit object recognition memory alterations in the second phase of the trial. **D** Social interaction test revealed no effect of genotype and sex in the frequency and/or duration of social interaction. **E**, **F** Genotype and sex did not affect immobility in both forced swim and tail suspension tests, respectively. **G** In the sucrose preference test, even though all animals showed an increased preference for the sucrose solution, the sucrose preference index revealed no genotype or sex effects. **H**, **I** There was no effect of the genotype and sex on the results of the nestled shredding and marble burying assays. For A-I wt: males: *n* = 8, females: *n* = 5; DDAH1-ko: males: *n* = 8, females: *n* = 5; two-way ANOVAs. Plots show mean ± SD center line and bottom and top lines; big circles represent the mean of males (light grey) and females (dark grey); small dots represent individual data points. *p < 0.05, **p < 0.01, ***p < 0.001 for significant genotype effects. Mouse tracking heatmaps show maximum (red) and minimum (blue) time spent at a certain point; the dotted white line depicts the centre zone in the open field and the objects in the novel object recognition and social interaction tasks

The open field arena and the elevated plus maze task were utilized to analyze locomotor activity and anxiety-like behavior. In the open field test, DDAH1-ko mice did not exhibit significant differences in the overall distance moved in the arena and the velocity of the movement compared to their wt counterparts, suggesting that the absence of DDAH1 has no effects on overall locomotor activity (Fig. 2A, Table S1). In addition, the time spent in the center zone, a variable associated with anxiety-like behaviors, also did not differ between genotypes (Fig. 2A, Table S1). Similar results were retrieved from the elevated plus maze test, with both genotypes spending a comparable amount of time in the closed and opened arms (Fig. 2B, Table S1). These data suggest that DDAH1 knock-out does not influence overall locomotor activity or innate anxiety-like behaviors.

Exploratory behavior and memory functions were assessed over 2 days in a novel object recognition test. In this task, animals are introduced to two unfamiliar objects, and due to the rodents’ innate preference for novelty, they are expected to explore them. In the second phase of the test, object recognition memory is assessed by analyzing the animal’s ability to evaluate a previously encountered object as familiar and manifest more exploration of the novel one. DDAH1-ko mice exhibited increased overall exploratory activity during the 2-day assessment by spending more time exploring the objects (Fig. 2C, *F* (1, 22) = 5.9, *p* = 0.024) with a significantly higher explorative activity in female DDAH1-ko mice (post-hoc comparison male vs. female DDAH1-ko: *p* = 0.004). However, when one of the objects was replaced by a novel one, both genotypes showed similarly increased preference for the novel object, indicating that recognition memory processes are not affected by the genotype (Table S1). Next, to further analyze whether the exploratory tendencies of DDAH1-ko mice would also appear in the form of social exploration, we performed a social interaction test in which animals were allowed to interact with an unfamiliar actor mouse. Nonetheless, in this case, DDAH1-ko mice did not exhibit altered social exploration but displayed similar frequency and duration of social contacts compared to controls (Fig. 2D, Table S1).

Stress-induced immobility was investigated using the tail suspension and forced swim tests. DDAH1-ko mice did not show significant differences in the amount of time being immobile compared to wt animals during both the forced swim test (Fig. 2E, Table S1) and tail suspension (Fig. 2F, Table S1), suggesting that DDAH1-ko animals are able to employ active-coping strategies in stressful situations.

Reward function was evaluated based on the sucrose preference test, which can discriminate between the motivational (wanting) and the hedonic (liking) components of a reward (Meyerolbersleben et al. 2020). RM-ANOVA analysis showed the expected effect of day (*F* (1, 88) = 95.705, *p* < 0.001), reflecting the switch from baseline water to sucrose during testing days, but no difference between genotypes nor interaction effects (Fig. 2G, Table S1), indicating that DDAH1-ko animals show intact liking and do not manifest anhedonic phenotypes. Similarly, an effect of day, attributed to increased consumption with the switch to sucrose (*F*(1,88) = 61.103, *p* < 0.001), but no genotype effect and interactions has been found when assessing absolute consumption, suggesting that the motivational component of the reward (wanting) is not influenced by the loss of DDAH1, as well (Table S1).

Finally, persistent and repetitive behaviors were examined using the nestled shredding and marble burying tests. DDAH1-ko mice exhibited unaltered nestled shredding activity (Fig. 2H, Table S1) and similar numbers of buried marbles compared to wt animals (Fig. 2I, Table S1).

Taken together, DDAH1-ko mice exhibited increased exploratory behavior when exposed to a novel object, while general locomotor activity and social exploration were unaltered. All further tested behaviors were unchanged.

### DDAH1-ko mice showed altered amphetamine sensitivity

**Fig. 3:**
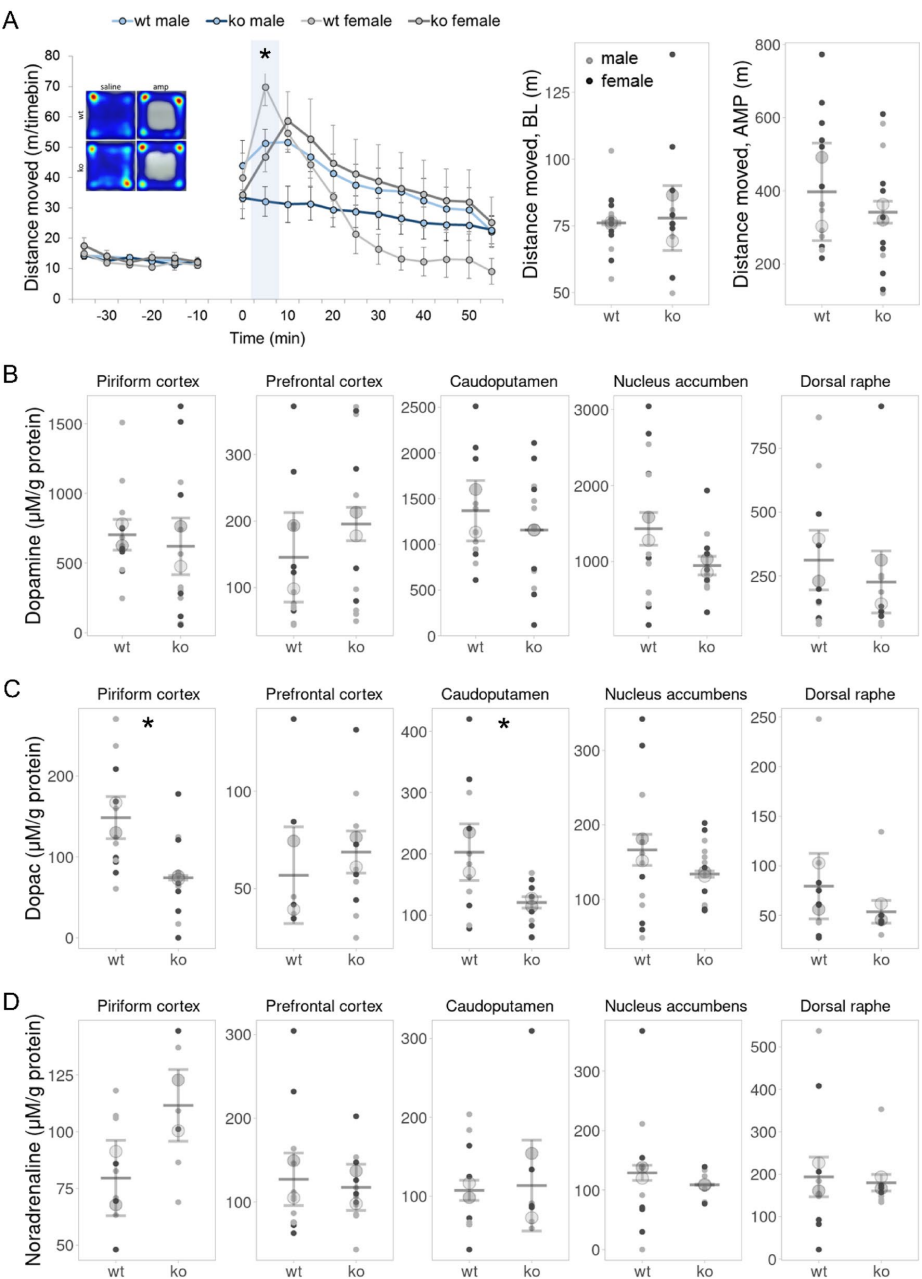
DDAH1-ko showed lower response to amphetamine. **A** Response to amphetamine was evaluated in amphetamine-induced activity test. Distance moved in baseline (BL) and after amphetamine injection (AMP) was independent from both gender and genotype. However, 5 min bin assessment revealed genotype-dependent differences in mice activity from 5 to 10 min; wt: males: *n* = 7, females: *n* = 5, DDAH1-ko: males: *n* = 8, females: *n* = 5; two-way ANOVAs and RM-AVOVA, respectively. One male wt animal was excluded due to an amphetamine overreaction and signs of cardiovascular dysfunction. **B**–**D** Analysis of neurotransmitters and metabolites implicated in amphetamine signaling. DDAH1-ko mice had lower DOPAC in the piriform cortex and caudoputamen, although DOPAC levels in prefrontal cortex, nucleus accumbens and dorsal raphe were independent from genotype and sex (**C**). Dopamine (**B**) and noradrenaline (**D**) levels showed no difference in all measured areas. Color code in crossed pathway heatmaps: blue (less time spent) to red (more time spent). For B–D wt: males: *n* = 7, females: n = 6, DDAH1-ko: males: *n* = 8, females: *n* = 6; two-way ANOVAs. Plots show mean ± SD center line and bottom and top lines; big circles represent mean of males (light grey) and females (dark grey); small dots represent individual data points. *p < 0.05, **p < 0.01, ***p < 0.001 for significant genotype effects. Mouse tracking heatmaps show maximum (red) and minimum (blue) time spent at a certain point

Dopaminergic neurons were shown to increase their firing in response to novel stimuli in several species and behavioral paradigms, highlighting that novelty is encoded by DA in the brain (Steinfels et al. 1983; Ljungberg et al. 1992; Horvitz et al. 1997; Schultz 2015). Thus, we next sought to determine whether *Ddah1* knock-out impacts brain dopaminergic tone through an amphetamine challenge paradigm. In line with the open field results, no genotype or sex effect was detected on baseline locomotor activity following saline injection (Fig. 3A, Table S1). However, after the administration of amphetamine, a significant effect of treatment has been observed (Fig. 3A, *F* (17, 357) = 19.322, *p* < 0.001). There was no main effect of genotype and sex in overall movement, including distance and velocity (Table S1), but a significant time x genotype interaction was observed (*F* (17, 357) = 2.62, *p* = 0.047), indicating that DDAH1-ko mice respond differently to amphetamine over time. Post-hoc tests of 5 min intervals after amphetamine injection revealed genotype- and sex-dependent differences in the kinetics of the early amphetamine response. Specifically, DDAH1-ko animals exhibited attenuated amphetamine sensitivity compared to wt (*F* (1, 21) = 8.414, *p* = 0.009), with females of both genotypes having a more robust response (*F* (1, 21) = 5.212, *p* = 0.033). Male DDAH1-ko had the least increase in their activity which over time remained constant. Stereotypic behavior has not been observed for any genotype or sex, and thus locomotor activity has not been compromised.

The observed behavioral alterations in DDAH1-ko animals suggested potential neurochemical deviations, and consequently, sensitive post-mortem HPLC was performed to determine region-specific levels of the major neurotransmitters and metabolites implicated in amphetamine signaling. DA is a key neurotransmitter implicated in amphetamine response (Wise 2004, 2008; Nutt et al. 2015), which also serves as a precursor for NOR synthesis in noradrenergic cells (Menniti and Diliberto 1989). However, genotype and sex effects have not been observed for DA and NOR levels in any investigated brain regions, including piriform cortex, prefrontal cortex, caudoputamen, nucleus accumbens and dorsal raphe (Fig. 3B, D, Table S2). Nonetheless, DOPAC—a major metabolite and indicator of DA catabolism—exhibited lower levels in piriform cortex and caudoputamen of DDAH1-ko animals (Fig. 3C, piriform cortex: *F* (1, 22) = 7.575, *p* = 0.012, caudoputamen: *F* (1, 23) = 8.013, *p* = 0.009).

In a nutshell, DDAH1-ko animals showed differences in the kinetics of an amphetamine challenge indicating a reduced sensitivity to amphetamine which is supported by the absence of stereotypic behavior and brain-specific alterations in dopamine homeostasis.

## Discussion

In this study, we sought to characterize the effects of a global DDAH1 knock-out on behavioral phenotypes relevant to neuropsychiatric disorders. We could show that (1) female DDAH1-ko mice exhibited increased exploratory behavior when exposed to novel objects; (2) DDAH1-ko animals of both sexes exhibited altered amphetamine response reflecting diminished sensitivity, which was accompanied by region-specific reduction of the main DA metabolite DOPAC.

Initially, we confirmed the lack of DDAH1 expression and enzymatic activity in the brain. DDAH1-ko animals exhibited normal physiological, morphological and developmental features. *Ddah1* consists of 9 exons and is located on the third chromosome in mice (Sayers et al. 2022). Currently, there are DDAH1 knock-outs models constructed either by first-exon deletion (Leiper et al. 2007; Zhao et al. 2021) or a fourth-exon deletion (Hu et al. 2011), with the latter being utilized in our study. One of the first-exon deletions was lethal in utero (Leiper et al. 2007; Breckenridge et al. 2010), whereas all the other knock-out models, including the one used in this study, had no detrimental effect on embryonic development. Generating knock-out constructs entails the risk of mutagenic and/or off-target effects and its precision highly depends on the particular genome engineering approach (Schulze and Lammers 2021). First-exon deletion may have interfered with neighboring genes essential for embryonic development, leading to impaired embryo implantation and death (Breckenridge et al. 2010).

To the best of our knowledge, this is the first study characterizing adult DDAH1-ko mice at the behavioral level. Since DDAH1 exerts its action via the NO pathway (Hu et al. 2011; Zhao et al. 2021), we assumed that our model might resemble behavioral phenotypes observed following the genetic deletion of the neuronal NOS (nNOS), the main supplier of NO in the brain (Tricoire and Vitalis 2012). Phenotypes observed in nNOS-ko animals include alterations in locomotor activity, social interactions, anxiety- and depressive-like behaviors, aggression and spatial memory (Nelson et al. 1995; Weitzdoerfer et al. 2004; Trainor et al. 2007; Wultsch et al. 2007; Tanda et al. 2009; Zhang et al. 2010; Gao and Heldt 2015). Hyperactivity and impaired spatial memory are the most robust phenotypes, while anxiety-like, social and aggressive behaviors remain still under debate. DDAH1-ko animals did not exhibit any of the above phenotypes; instead, they showed increased exploratory behavior toward novel objects. Novel stimuli create approach–avoidance conflict by stimulating both the exploration essential to survival (e.g., food-seeking) and avoidance of potentially threatening situations (Montgomery 1955; Powell et al. 2004). Still, exploration or novelty-seeking is an essential aspect of behavior and has been well-documented, for example, in the preference for a novel object over a familiar object (Ennaceur and Delacour 1988). Novelty-seeking has been implicated as a predictive variable for animal drug-taking tendencies (O’Connor et al. 2021) and connected to clinical substance abuse populations (Kovács et al. 2022). Observations in nNOS-ko animals, such as frequent crossings and increased time in the center zone of the open field as well as increased time in the open arms of the elevated plus maze, could be interpreted as a form of exploratory behavior (Weitzdoerfer et al. 2004). Though, normal novel object and environment exploration has also been reported for these models (Tanda et al. 2009). Overall, further investigations on spatial and/or object exploration in NOS models are essential to dissect novelty-seeking from locomotor hyperactivity.

Several reasons can account for the limited behavioral alterations in our model. Due to its indirect effect on NO regulation, DDAH1 knock-out can be expected to show more subtle effects than direct targeting of NO synthesis via NOS. In addition, alternative pathways could have eliminated ADMA, and thus minimized the behavioral outcome. Despite its questionable role in ADMA metabolism (Hu et al. 2011), DDAH2 overexpression (Pope et al. 2009; Torondel et al. 2010) and knock-out (Wang et al. 2007; Pope et al. 2009) were shown to alter NO synthesis, presumably by ADMA-independent mechanism, and thus this enzyme could potentially counterbalance the absence of its second isoform. Trans-amination by alanine-glyoxylate aminotransferase 2 (AGXT2) or increased excretion in urine (Rodionov et al. 2014; Oliva-Damaso et al. 2019) could also be involved in ADMA elimination. Although ADMA is only partially eliminated through these pathways and the expression level of AGXT2 in the brain is very low, involvement in these routes cannot be excluded. Finally, DDAH1 is shown to bind to and increase Ras activity which in turn activates the protein kinase B (PKB/AKT) pathway (Zhang et al. 2011). This pathway has been associated with neurological and psychiatric disorders (Saudou et al. 1998; Ikeda et al. 2004; Griffin et al. 2005) and particularly with anxiety, spatial and contextual memory and fear extinction (Wong et al. 2020). We realize that not assessing those alternative pathways is a limitation of our study.

The second main finding of our study is that DDAH1-ko mice showed reduced sensitivity to amphetamine. The acute amphetamine response phenotype is viewed as an endophenotype for psychiatric disorders with DA system alterations, such as schizophrenia and attention deficit hyperactivity disorder (ADHD). While basic DA theories view ADHD and schizophrenia as problems on opposite sides of the DA spectrum, a complex dopamine model has been used to suggest that schizophrenia and ADHD could result from the same tonic DA changes (Yanofski 2010). Indeed, genetic variation associated with the euphoric effects of d-amphetamine is associated with the risk for both disorders (Hart et al. 2014). Of note, in line with our data, females have been shown to have a greater response to psychomotor stimulants (Becker et al. 2001; Van Swearingen et al. 2013).

Amphetamine acts through the DA transporter (DAT), a membrane protein responsible for the reuptake of synaptic DA, by triggering DA outflow through DAT-mediated reverse transport and redistribution of DA from its vesicular localization to the cytoplasm (Mortensen and Amara 2003; Howell and Kimmel 2008; Zhu and Reith 2008). Given the critical role of DAT in amphetamine’s mechanism of action, it can be assumed that the rate of neurotransmitter clearance, cellular transport capacity and normal receptor stimulation can be altered upon modification of DAT expression. In a zebrafish nNOS-ko model, DAT mRNA expression had markedly increased, suggesting a corresponding increase also in DAT transporter (Penglee et al. 2021). Indeed, NO influences DA release and uptake processes (Kiss 2000; Salum et al. 2016), and more specifically, it has been shown to exert an inhibitory effect on the DAT (Kiss et al. 2004). Therefore, NO reduction due to DDAH1 loss could enhance DAT expression, balance DA reuptake, and reduce amphetamine sensitivity during the initial response phase. The observed reduced DOPAC levels within the caudoputamen in DDAH1-ko animals underpin our behavioral observation, as DOPAC is formed intraneuronal by degradation of unbound cytoplasmic DA, and thus provides an index of the size of the cytoplasmic pool of DA (Robinson and Camp 1991). Once more, the expected reduction of NO signaling in the DDAH1-ko model may directly contribute to reduced dopamine metabolism to DOPAC through its modulatory effects on the monoamine oxidase activity (Carreño Gutiérrez et al. 2020).

Despite the previous report of reduced plasma NO levels in the DDAH1-ko mice (Hu et al. 2011), the major limitation of our study is the lack of experimental evidence for the impact of DDAH1 loss on the brain or brain-region-specific changes in NO levels. In DDAH1-ko rats a reduction of NO in whole brain homogenate has been shown (Zhao et al. 2021). In addition, Freudenberg et al. associated hippocampus-specific alterations in NO levels with social and working memory impairments but not with other phenotypes, highlighting that brain-region-specific effects in the machinery regulating NO synthesis may govern the manifestation of specific behaviors (Freudenberg et al. 2021). Therefore, assessing global and region-specific NO levels in the brain of DDAH1-ko mice and accordingly targeted DDAH1 activity modulation in different brain regions should be essential next steps to increase our understanding of DDAH1 function within the CNS.

To conclude, DDAH1 influences behavior and the dopamine system, most probably via the NO pathway. This study sets the basis for further experimentation in this field.

## Supplementary Information

Below is the link to the electronic supplementary material.

Supplementary file1 (XLSX 26 kb)

Supplementary file2 (XLSX 18 kb)

## Data Availability

All data generated or analysed during this study are included in this published article and its supplementary information files.
